# Exploring Gut Microbiota Alterations with Trimethoprim-Sulfamethoxazole and Dexamethasone in a Humanized Microbiome Mouse Model

**DOI:** 10.3390/microorganisms12051015

**Published:** 2024-05-17

**Authors:** George B. H. Green, Alexis N. Cox-Holmes, Olivia Backan, Olivia Valbak, Anna Claire E. Potier, Dongquan Chen, Casey D. Morrow, Christopher D. Willey, Braden C. McFarland

**Affiliations:** 1 Department of Cell, Developmental and Integrative Biology, Birmingham, AL 35294, USA; 2 Undergraduate Cancer Biology Program, Birmingham, AL 35294, USA; 3 Department of Genetics, Birmingham, AL 35294, USA; 4 Department of Radiation Oncology, Heersink School of Medicine, University of Alabama at Birmingham, Birmingham, AL 35233, USA

**Keywords:** microbiome, trimethoprim, sulfamethoxazole, dexamethasone, glioblastoma

## Abstract

Along with the standard therapies for glioblastoma, patients are commonly prescribed trimethoprim-sulfamethoxazole (TMP-SMX) and dexamethasone for preventing infections and reducing cerebral edema, respectively. Because the gut microbiota impacts the efficacy of cancer therapies, it is important to understand how these medications impact the gut microbiota of patients. Using mice that have been colonized with human microbiota, this study sought to examine how TMP-SMX and dexamethasone affect the gut microbiome. Two lines of humanized microbiota (HuM) Rag1^−/−^ mice, HuM1Rag and HuM2Rag, were treated with either TMP-SMX or dexamethasone via oral gavage once a day for a week. Fecal samples were collected pre-treatment (pre-txt), one week after treatment initiation (1 wk post txt), and three weeks post-treatment (3 wk post txt), and bacterial DNA was analyzed using 16S rRNA-sequencing. The HuM1Rag mice treated with TMP-SMX had significant shifts in alpha diversity, beta diversity, and functional pathways at all time points, whereas in the HuM2Rag mice, it resulted in minimal changes in the microbiome. Likewise, dexamethasone treatment resulted in significant changes in the microbiome of the HuM1Rag mice, whereas the microbiome of the HuM2Rag mice was mostly unaffected. The results of our study show that routine medications used during glioblastoma treatment can perturb gut microbiota, with some microbiome compositions being more sensitive than others, and these treatments could potentially affect the overall efficacy of standard-of-care therapy.

## 1. Introduction

Glioblastoma (GBM) is the most frequently diagnosed malignant primary brain tumor [1,2,3,4]. The World Health Organization (WHO) classifies GBM as a grade 4 tumor, which is molecularly confirmed by the presence of *IDH* wild-type, *TERT* promoter alterations, chromosome 7/10 gain/loss, and *EGFR* alterations [5]. The standard of care includes maximal safe surgical resection, chemotherapy, radiation, and a tumor-treating field device (Optune Gio^®^) [6,7], yet long-term survival is very poor, with the 5-year survival being only 5% [8].

Medications commonly used along with standard care therapies for patients with GBM include dexamethasone and trimethoprim-sulfamethoxazole (TMP-SMX) [9,10,11]. Dexamethasone is a potent glucocorticoid steroid drug that acts as an anti-inflammatory agent or immunosuppressant by increasing glucogenesis and blocking inflammatory mediators [11,12]. Dexamethasone is commonly used to reduce cerebral edema, a side effect of standard care treatment for patients with GBM [12,13]. In recent studies, dexamethasone has been linked to shortened survival and decreased immunotherapy efficacy in GBM models [12,13,14]. TMP-SMX is a broad-spectrum combination antibiotic [15]. Sulfamethoxazole inhibits the synthesis of dihydrofolic acid by acting as a competitor of PABA and inhibiting the enzyme dihydropteroate synthase [15]. Trimethoprim inhibits thymidine and DNA synthesis through its direct competition with the enzyme dihydropteroate synthase [15]. TMP-SMX is commonly prescribed to patients with GBM to prevent numerous infections, including *pneumocystis jiroveci* pneumonia, during treatment cycles [9]. It is unclear how TMP-SMX and dexamethasone disrupt the microbiome in patients with GBM.

The gut microbiota and its metabolites can generate immunological and cellular pathways which eradicate invading pathogens and stimulate an immune response, the latter being an important mechanism to prevent cancer formation [16]. Recent reports indicate that the gut microbiome can influence the response to therapy in numerous cancers, and that antibiotic treatment during immunotherapy attenuates overall survival [17,18,19,20,21,22,23,24]. The gut microbiota also can affect the efficiency of chemotherapeutic drugs and various methods of cancer treatment [16,25,26].

To understand how medications commonly administered to patients with GBM could disrupt the gut microbiota, we utilized a humanized microbiome (HuM) mouse model where mice intestines are colonized by microbial species from healthy human donors [27,28]. In this study, we examined the changes in the microbiota in response to either dexamethasone or TMP-SMX, using two humanized microbiome (HuM) lines (HuM1 and HuM2). In order to further comprehend the effect of TMP-SMX and dexamethasone on the microbiota, B6.Rag1^−/−^ mice, which lack B and T cells, were utilized to focus on the effect of these medications without the impact of the adaptive immune system shaping the microbiome, as this could interfere with overall colonization and starting microbial composition. Furthermore, patients with GBM at the time of diagnosis are severely immunosuppressed, even before radiation and chemotherapy treatment [29], thus making the Rag1^−/−^ mouse strain a comparable immune environment to study the effects of TMP-SMX and dexamethasone on the microbiome.

We found that treatment with either TMP-SMX or dexamethasone led to shifts in the microbial composition of both the HuM mouse lines. However, the degree of microbiota disruption varied between the HuM lines, suggesting that some microbial species may be more sensitive to the TMP-SMX or dexamethasone treatment. Specifically, we found that the HuM1Rag mice had a more significant disruption, which did not return to pre-treatment abundances and diversity. This indicated that for certain microbiome compositions, common treatments may infer long-term changes in the microbiome and have implications for shortened survival and immunotherapy efficacy in patients. Ultimately, understanding the microbiome disruptions following the dexamethasone or TMP-SMX treatment and recovery could lead to preferable therapeutic options in treating the side effects of standard-of-care (chemotherapy and radiotherapy) treatment for patients with GBM.

## 2. Materials and Methods

### 2.1. Reagents

Trimethoprim and sulfamethoxazole were purchased from Sigma-Aldrich (St. Lewis, MO, USA). Dexamethasone was purchased from MP Biomedicals, LLC (Solon, OH, USA). Oraplus vehicle suspension solution was purchased from Amazon.

### 2.2. Mice

The cryopreserved cecal samples of two lines of the humanized microbiome (HuM) mouse breeders (HuM1 and HuM2) from previously published research were used for fecal transplantation into gnotobiotic mice [27,30]. Gnotobiotic mice (B6.Rag1^−/−^), which are deficient in mature B and T cells, were obtained from the UAB Gnotobiotic Core. Rag1^−/−^ gnotobiotic mice were given 100–200 μL of cecal matter via oral gavage resulting in two distinctive humanized microbiome mouse lines: HuM1Rag and HuM2Rag. The mice were bred and the progeny were used in all experiments for the study. At older than 6 weeks of age, the mice were administered vehicle (Oraplus; HuM1Rag *n* = 5; HuM2Rag *n* = 5), TMP-SMX (40 mg/kg trimethoprim and 200 mg/kg sulfamethoxazole; HuM1Rag *n* = 7; HuM2Rag *n* = 6), or dexamethasone (10 mg/kg; HuM1Rag *n* = 6; HuM2Rag *n* = 6) via oral gavage once a day for seven days. Both male and female mice were used in each treatment group. Fecal samples were collected pre-treatment (Pre-txt), one week following initial treatment (1 wk post txt), and three weeks post-treatment (3 wk post txt). Fecal samples were collected and then stored at −20 °C.

### 2.3. Sample Preparation for High-Throughput Sequencing

Total DNA was isolated from fecal samples utilizing the Quick DNA Fecal/Soil Microbe Miniprep (Cat# D6010, ZYMO Research, Tustin, CA, USA) per the manufacturer’s instructions. Purified DNA was subjected to quantification and purity assessment via an Epoch microplate spectrophotometer (BioTek Instruments, Winooski, VT, USA).

### 2.4. High-Throughput Sequencing

High-throughput amplicon sequencing was performed utilizing Illumina MiSeq with the 250 bp paired-end kits (Illumina, Inc., San Diego, CA, USA) and via targeting the V4 hypervariable region of the bacterial 16S rRNA gene. The obtained sequences were demultiplexed and formatted in FASTQ. Raw sequence files were submitted to the National Center for Biotechnology Information (NCBI) Sequence Read Archive (SRA) under the following BioProject number PRJNA1100598; this includes the TMP-SMX-treated HuM1 and HuM2 mice, dex-treated HuM1 and HuM2 mice, and vehicle-treated HuM1 and HuM2 mice. The subgroups were designated as follows: HuM1Rag Pre-txt (*n* = 17), HuM1Rag 1 wk post txt (*n* = 7), and HuM1Rag 3 wk post txt (*n* = 7). Additionally, the subgroups for the HuM2Rag mice were similarly labeled: HuM2Rag Pre-txt (*n* = 17), HuM2Rag 1 wk post txt (*n* = 6), and HuM2Rag 3 wk post txt (*n* = 6).

### 2.5. Taxonomic Assignment and Distribution

The taxonomic profiles of HuM1 and HuM2 treated with TMP-SMX, dexamethasone, or vehicle were established via QIIME2 (2023.5) [31]. The initial FASTQ sequence files were imported into QIIME2 (2023.5) [31] via the “qiime tools import” utilizing the cassava 1.8 paired-end demultiplexed FASTQ file format (CasavaOneEightSingleLanePerSampleDirFmt). Sequence files were quality-checked using “qiime demux summarize” input. After that, denoising methods were implemented utilizing DADA2 (q2-dada2 denoize-paired) [32]. The DADA2 output was summarized via the “qiime feature-table summarize” command (Supplementary Data S1). Representative sequences were generated via the q2-feature-table tabulate-seqs input. The mafft program (q2-alignment) aligned amplicon sequence variants (ASVs) [33] and the output was then used via fasttree2 (q2-phylogeny) to generate the phylogeny [34] using the default building methods. To generate alpha diversity, Faith’s phylogenetic diversity [35], beta diversity, unweighted UniFrac [36], Jaccard distance, Bray–Curtis dissimilarity, principal coordinate analysis (PCoA), Simpson [37], and Shannon [38] metrics were used via “core–metrics–phylogenetic” command via “q2-diversity plugin”. The samples were rarefied to 5320 sequences per sample minimum. ASVs were assigned via the q2-feature-classifier command [39] plugin utilizing “classify-sklearn” against the silva-138-99-nb-classifier [40]. The taxonomy was then collapsed via “qiime taxa collapse” into table format. Taxonomic identities were determined based on their assignment through the SILVA v138 database, using Quantitative Insights into Microbial Ecology (QIIME2, v2023.5), and graphed using R (ggplot package v. 3.4.4) and PhyloSeq (v1.41.1). A detailed list of the ASVs can be found in Supplementary Data S2. (for clarity “Unknown” and “Other” were filtered out). Beta diversity metrics were determined using the taxonomy data. PCoA plots depict relationships: PCoA1 vs. PCoA2 was calculated using the Bray–Curtis metrics, and PCoA1 vs. PCoA2 was calculated using weighted UniFrac. Ellipses were added based on default settings in MicrobiotaProcess (v1.6.6) with a confidence level of 0.9. Alpha-diversity measurements (observed ASVs, Shannon diversity index, and Simpson’s index) were obtained for pre-treatment groups based on QIIME2 (v2023.5) output and plotted using phyloseq (v1.41.1) (for Genus level ASVs for clarity “Unknown” and “Other” were filtered out).

### 2.6. Predicted Functional Analysis

Phylogenetic Investigation of Communities by Reconstruction of Unobserved States (PiCRUSt2, v2.5.2) [41] was used to determine predicted functional profiles of the gut microbiota across the humanized mouse microbiota samples (HuM1Rag and HuM2Rag). The command “picrust2_pipeline.py” outputted the hidden-state prediction of genomes, metagenome predictions, sequence placement, pathway-level predictions, and Nearest Sequenced Taxon Index (NSTI) values. Descriptions were added to the metagenome predictions via the “add_descriptions.py” command, which describes each functional capacity [41]. The Kyoto Encyclopedia of Genes and Genomes (KEGG) functional profiles were obtained utilizing ggpicrust2 [42], which provided KEGG profile descriptions. Differential functional abundances between HuM1Rag and HuM2Rag were determined via ggpicrust2 [42,43]. The top pathways were plotted based on adjusted *p*-value, barplots represent functional abundance, and divergent barplots represent log2fold changes.

### 2.7. Read Quality and Sample Statistics and Taxonomic Distribution across All Samples

The paired-end Illumina MiSeq analysis of the V4 segment of the 16S rRNA gene amplicons generated a raw sequence count and yielded 3,413,174 reads following dada2 quality checking. A total of 1640 observed ASVs were identified after the QIIME2 (v2023.5) quality filtering process (Supplementary Data S1). The observed taxonomic distribution is presented in Supplemental Data S2. All the bacteria discovered through QIIME2 (v2023.5) across all the samples are displayed in Supplemental Figure S1. The most abundant phyla seen across all the samples were Firmicutes, Actinobacteriota, Bacteroidota, and Proteobacteria.

## 3. Results

### 3.1. HuM1Rag and HuM2Rag Are Unique Microbiome Lines

**Figure 1:**
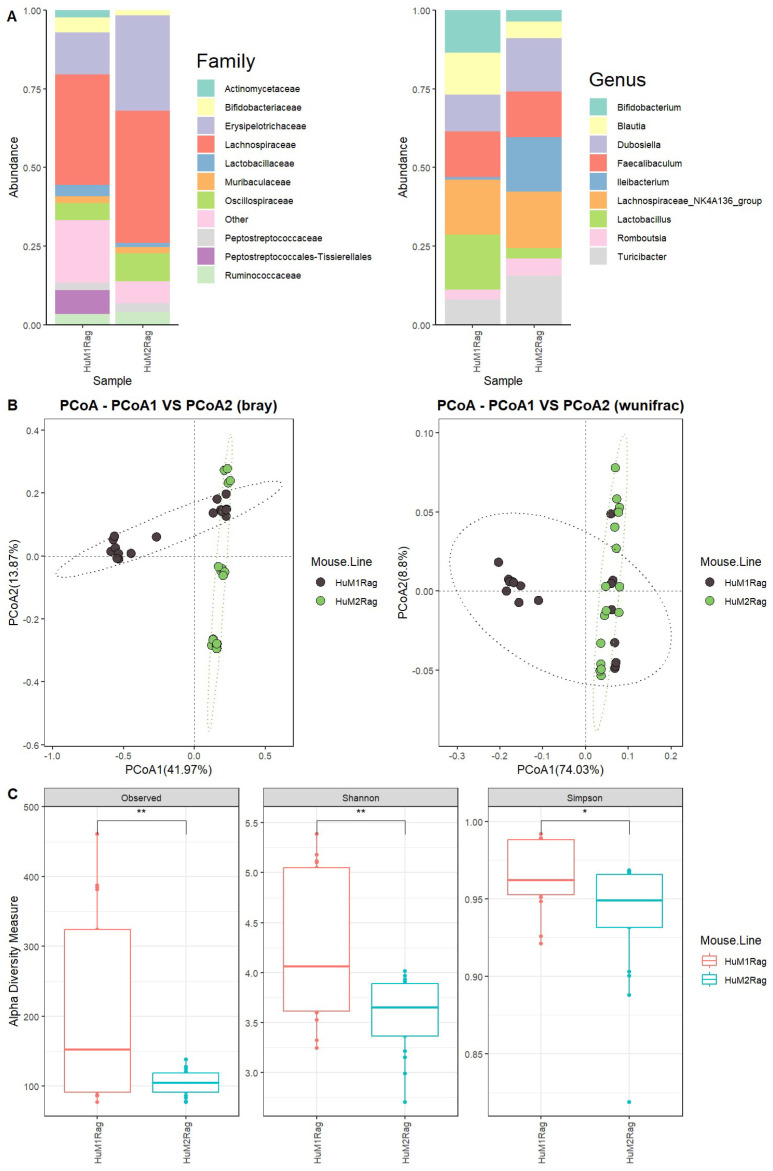
HuM1Rag and HuM2Rag are unique microbiome lines. (**A**) The relative abundance of the top 10 taxa at the family level and Genus level across HuM1Rag and HuM2Rag pre-treatment. (**B**) Beta diversity was determined utilizing the Bray-Curtis and weighted UniFrac metrics for the pre–treatment groups. (**C**) Alpha-diversity measurements (observed ASVs, Shannon diversity index, and Simpson’s index) were obtained for the pre-treatment groups.

We first evaluated the microbiome difference between HuM1Rag and HuM2Rag at the baseline (pre-treatment) to confirm that they are two distinct microbiome lines with regard to taxonomic distribution and diversity. Firmicutes were the dominant taxon across the HuM1Rag and HuM2Rag pre-treatment groups (Supplemental Figure S1). Additionally, HuM1Rag (Supplemental Figure S2) and HuM2Rag (Supplemental Figure S3) mice were administered the vehicle for controls. HuM2Rag mice were revealed to have an increased abundance of the family Lachnospiraceae (~42), and Erysipelotrichaceae (~30%) compared to the HuM1Rag mice (~35%) and (~13%) (Figure 1A). Additionally, the HuM1Rag mice had a population of Peptostreptococcales-Tissierellales (~8%) which was not observed in the HuM2Rag mice. At a Genus level, *Lachnospiraceae_NK4A136_group* primarily composed both the HuM1Rag (~18%) and HuM2Rag (~18%) mice (Figure 1A). The HuM1Rag mice had a higher abundance of *Lactobacillus* (~17%), *Bifidobacterium* (~13%), and *Blautia* (13%). In contrast, the HuM2Rag mice had higher abundances of *Ileibacterium* (~17%), *Dubosiella* (~17%), and *Turicibacter* (~16%) (Table 1).

Beta diversity was determined utilizing the Bray–Curtis and weighted UniFrac metrics across all the HuM1Rag and HuM2Rag mice pre-treatment samples. The HuM1Rag and HuM2Rag mice displayed distinct clustering between mouse lines (Figure 1B). Although there was a slight overlap in clustering, PERMANOVA statistics supported significant dissimilarity among the sample groups (R^2^ = 0.2), with *p* values (<0.05); however, PERMDISP revealed a significant dispersion of the samples (*p* < 0.05), which confirms significant differences between the HuM1Rag and HuM2Rag mice. Additionally, the effect of mouse line and cage number was tested, which resulted in significant dissimilarity among the sample groups (R^2^ = 0.34), with *p* values (<0.05), and PERMDISP revealed no significant dispersion of the samples (*p* > 0.05). Overall, these data confirm that the HuM1Rag and HuM2Rag microbiome mice are distinct microbiome lines with regard to microbial taxonomic distribution and diversity. With regard to alpha diversity, the observed ASVs revealed significant (*p* < 0.05) differences between the HuM1Rag and HuM2Rag mice (Figure 1C). Additionally, both the Shannon diversity and Simpson diversity revealed a significant difference (*p* < 0.05) between the HuM1Rag and HuM2Rag mice sample groups (Figure 1C). This indicates that the HuM1Rag and HuM2Rag mice have differences in microbial composition.

### 3.2. Predicted Functional Analysis between HuM1Rag and HuM2Rag Mice Pre–Treatment

In order to assess potential functional differences between the HuM1Rag and HuM2Rag mice pre-treatment, PiCrust2 analyses were performed. For the HuM1Rag mice, there was a significant upregulation in the KEGG pathways associated with human diseases, cancer, and cardiovascular and infectious diseases. Additionally, the HuM1Rag mice had an upregulation in energy, lipid, cofactors, vitamins, terpenoids, and polyketide metabolism. Lastly, HuM1Rag had an increase in pathways related to cell growth and death, transport and catabolism, circulatory system, and digestive system-related processes. In contrast, the HuM2Rag mice were revealed to have an upregulation in membrane transport, carbohydrate metabolism, and amino acid metabolism (Supplemental Figure S4). These data suggest that there are differences in the predicted functional pathways of the microbiota of the HuM1Rag and HuM2Rag mice.

#### 3.2.1. TMP-SMX Significantly Altered the Microbiome of HuM1Rag Mice

**Figure 2:**
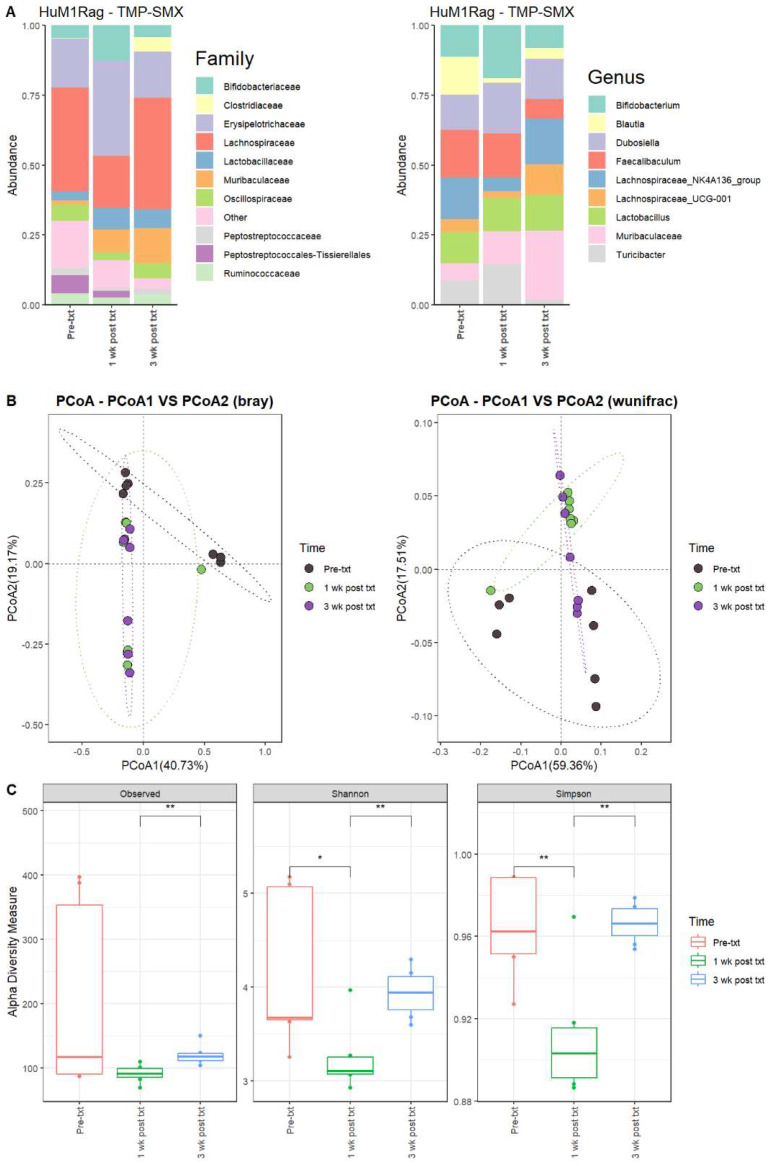
TMP-SMX significantly altered the microbiome of the HuM1Rag mice. (**A**) The relative abundance of the top 10 taxa at the family level and Genus level across the HuM1Rag TMP–SMX samples: Pre–txt, 1 wk post txt, and 3 wk post txt. (**B**) Beta diversity was determined utilizing the Bray–Curtis and weighted UniFrac metrics for the indicated groups. (**C**) Alpha-diversity measurements (observed ASVs, Shannon diversity index, and Simpson’s index) were obtained for the indicated groups.

Next, we determined the microbial changes following TMP-SMX treatment. For the HuM1Rag mice one week post-treatment with TMP-SMX (1 wk post txt), there were significant overall shifts in the microbial composition. Firmicute members decreased, while Actinobacteriota and Bacteroidota showed an increase in overall abundance. At the family level, pre-treatment (Pre-txt) was characterized via a large abundance of Lachnospiraceae (~37%) members. However, 1 wk post txt, there was a significant drop in Lachnospiraceae members (~19%) (Figure 2A). Moreover, at the Genus level, *Faecalibaculum* (~17%), *Lachnospiraceae_NK4A136_group* (~15%), and *Blautia* (~14%) dominated the microbial composition (Figure 2A). At 1 wk post txt, there was a decrease in *Faecalibaculum* (~15%), *Lachnospiraceae_NK4A136_group* (~5%), and *Blautia* (~2%), accompanied via an increase in *Bifidobacterium* (~19%), *Dubosiella* (18%), *Turicibacter* (~15%), and Muribaculaceae (~12%) (Table 2). This supports that TMP-SMX altered the microbial composition in comparison to the original microbial composition pre-treatment in the HuM1Rag mice.

#### 3.2.2. HuM1Rag Mice Did Not Recover to Original Microbial Composition Post-Treatment with TMP-SMX

At three weeks post-treatment (3 wk post txt), Muribaculaceae (~25%) became the dominant member of the HuM1Rag microbiota. This was followed by *Lachnospiraceae_NK4A136_group* (~16%) and *Dubosiella* (~14%) (Figure 2A; Table 2). Looking at the beta diversity, HuM1Rag Pre-txt, 1 wk post txt, and 3 wk post txt displayed distinct clustering (Figure 2B). PERMANOVA statistics supported the significant dissimilarity among the sample groups (R^2^ = 0.25), with *p* values (<0.05), and PERMDISP revealed no significant dispersion of the samples (*p* > 0.05). Additionally, the majority of the 1 wk post txt and 3 wk post txt samples clustered together away from the pre-txt samples, indicating that the microbial composition of some HuM1Rag mice did not recover from the TMP-SMX treatment. Alpha diversity observed ASVs revealed significant (*p* < 0.05) differences between the 1 wk post txt and 3 wk post txt HuM1Rag mice (Figure 2C). The Shannon diversity and Simpson diversity revealed a significant difference (*p* < 0.05) between the pre-txt and 1 wk post txt, and 1 wk post txt and 3 wk post txt HuMRag1 mice. (Figure 2C). This indicates that certain microbial groups were altered following the TMP-SMX treatment, and have not returned to pre-treatment abundances.

#### 3.2.3. TMP-SMX Altered the Microbiome of HuM2Rag Mice

**Figure 3:**
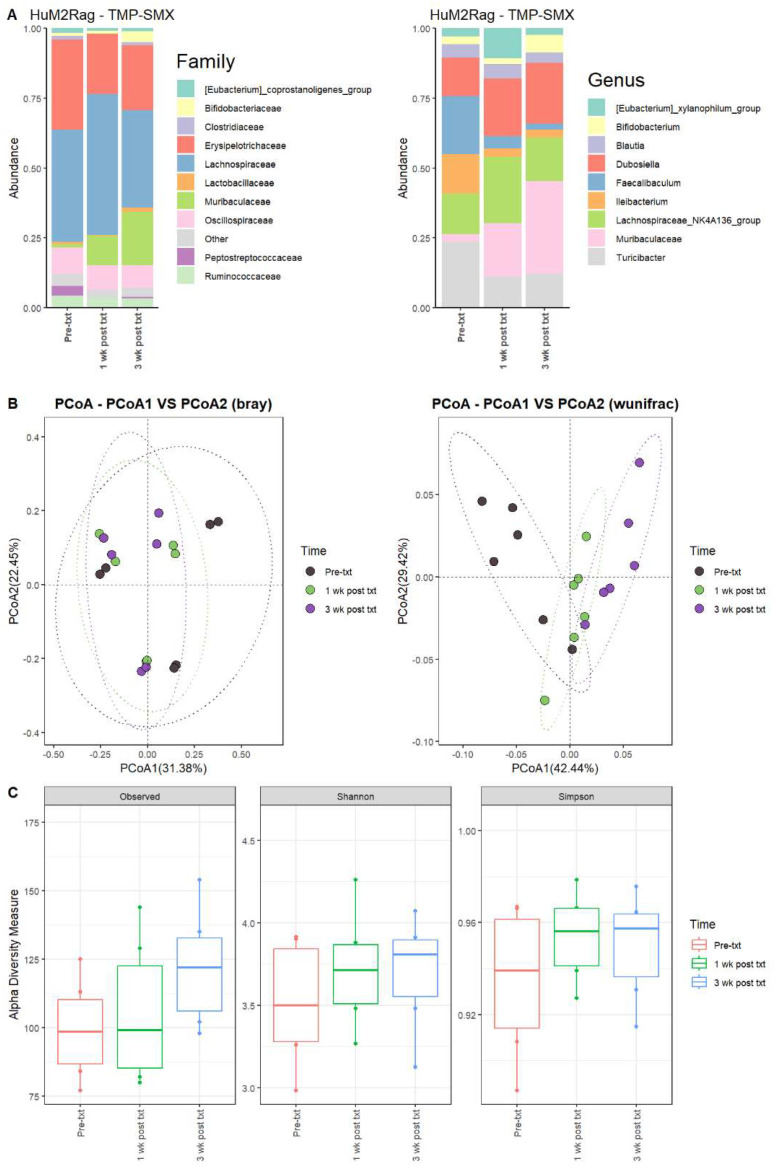
TMP-SMX altered the microbiome of HuM2Rag mice. (**A**) The relative abundance of the top 10 taxa at the family level and Genus level across the HuM2Rag TMP-SMX samples: Pre-txt, 1 wk post txt, and 3 wk post txt. (**B**) Beta diversity was determined utilizing the Bray–Curtis and weighted UniFrac metrics for the indicated groups. (**C**) Alpha-diversity measurements (observed ASVs, Shannon diversity index, and Simpson’s index) were obtained for the indicated groups.

For the HuM2Rag mice 1 wk post txt with TMP-SMX, the Firmicute members decreased, while Bacteroidota showed an increase in overall abundance (Table 3). Pre-txt was characterized via a large abundance of Lachnospiraceae (~40%) family members, and 1 wk post txt, there was an increase in Lachnospiraceae members (~50%) (Figure 3A). At the Genus level, *Turicibacter* (~24%), *Faecalibaculum* (~21%), *Lachnospiraceae_NK4A136_group* (~14%), and *Ileibacterium* (~14%) dominated the microbial composition. After 1 wk post txt, there was a decrease in *Turicibacter* (~11%), *Faecalibaculum* (~4%), and *Ileibacterium* (~3%), accompanied by an increase in *Lachnospiraceae_NK4A136_group* (~24%), *Dubosiella* (~21%), and *Muribaculaceae* (~19%) (Table 3). These results suggest that certain microbial groups were altered following TMP-SMX treatment in the HuM2Rag mice.

#### 3.2.4. HuM2Rag Mice Microbiome Recovered from TMP-SMX Treatment

At three weeks post-treatment (3 wk post txt), Muribaculaceae (~33%) became the dominant member of the HuM2Rag microbiota, followed by *Dubosiella* (~22%) (Figure 3A; Table 3). Regarding beta diversity, the HuM2Rag mice resulted in distinct clustering across the treatment groups, and PERMANOVA statistics supported the significant dissimilarity among the sample groups (R^2^ = 0.21), with *p* values (<0.05), and PERMDISP revealed no significant dispersion of the samples (*p* > 0.05) (Figure 3B). These data demonstrate that the HuM2Rag microbiota is much more static following the treatment with TMP-SMX compared to the dynamic changes observed in the HuM1Rag mice. Additionally, all 1 wk post txt and 3 wk post txt samples clustered together alongside the pre-txt samples, indicating that the microbial composition of the HuM2Rag mice did not change to the same extent observed in the HuM1Rag mice. Interestingly, in contrast to the HuM1Rag mice treated with TMP-SMX, the HuM2Rag mice revealed no differences in alpha diversity across the treatment groups (Figure 3C). These data suggest that the microbiota recovered from the TMP-SMX treatment in the HuM2Rag mice.

#### 3.2.5. Predicted Functional Analysis of HuM1Rag Revealed Significant Pathways Affected by TMP-SMX Treatment

**Figure 4:**
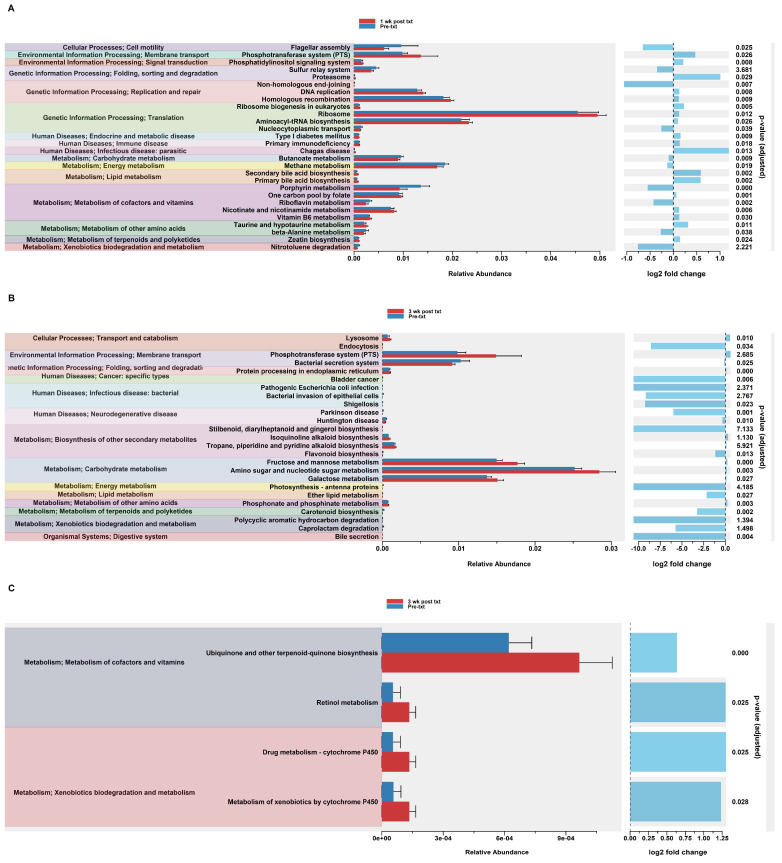
Predicted functional analysis of HuM1Rag and HuM2Rag revealed pathways affected by the TMP-SMX treatment. Predicted functional pathways were determined via PICRUSt and displayed are the differential abundance of the predicted functional pathways. (**A**) HuM1Rag Pre-txt vs. 1 wk post txt; (**B**) HuM1Rag Pre-txt vs. 3 wk post txt; (**C**) HuM2Rag Pre-txt vs. 3 wk post txt.

In order to assess potential functional differences following the TMP-SMX treatment, PiCrust2 analyses were performed. PiCrust2 analysis revealed a significant difference across the predicted KEGG pathways at 1 wk post txt in HuM1Rag (Figure 4A). Among these pathways, there was a significant upregulation across membrane transport, Replication and Repair, Translation, and carbohydrate metabolism. Additionally, a significant upregulation in primary and secondary bile acid synthesis was observed. At 3 wk post txt in the HuM1Rag mice, a significant upregulation was observed in carbohydrate metabolism, associated with fructose and mannose metabolism, amino sugar and nucleotide sugar metabolism, and galactose metabolism (Figure 4B). Additionally, a significant down-regulation in the pathways associated with cancer, infectious diseases, and neurodegenerative diseases was observed. This demonstrates that there are potentially numerous functional pathways altered in the HuM1Rag mice by the TMP-SMX treatment.

#### 3.2.6. Predicted Functional Analysis from TMP-SMX Treatment of HuM2Rag Revealed Fewer Pathways Altered when Compared to HuM1Rag Mice

Notably, the PiCrust2 analysis in the HuM2Rag mice revealed only a singular significant difference in the ether lipid metabolism pathway at 1 wk post txt. At 3 wk post txt, there was a significant increase in cofactor and vitamin metabolism (Retinol and Ubiquinone, and other terpenoid-quinone biosynthesis), and xenobiotic biodegradation (by cytochrome P450), and drug metabolism (cytochrome P450) (Figure 4C). Overall, the PiCrust2 analyses revealed that the HuM1Rag mice exhibited many more predicted pathway changes following the TMP-SMX treatment than the HuM2Rag mice.

#### 3.2.7. Dexamethasone Significantly Alters the Microbiome of HuM1Rag

**Figure 5:**
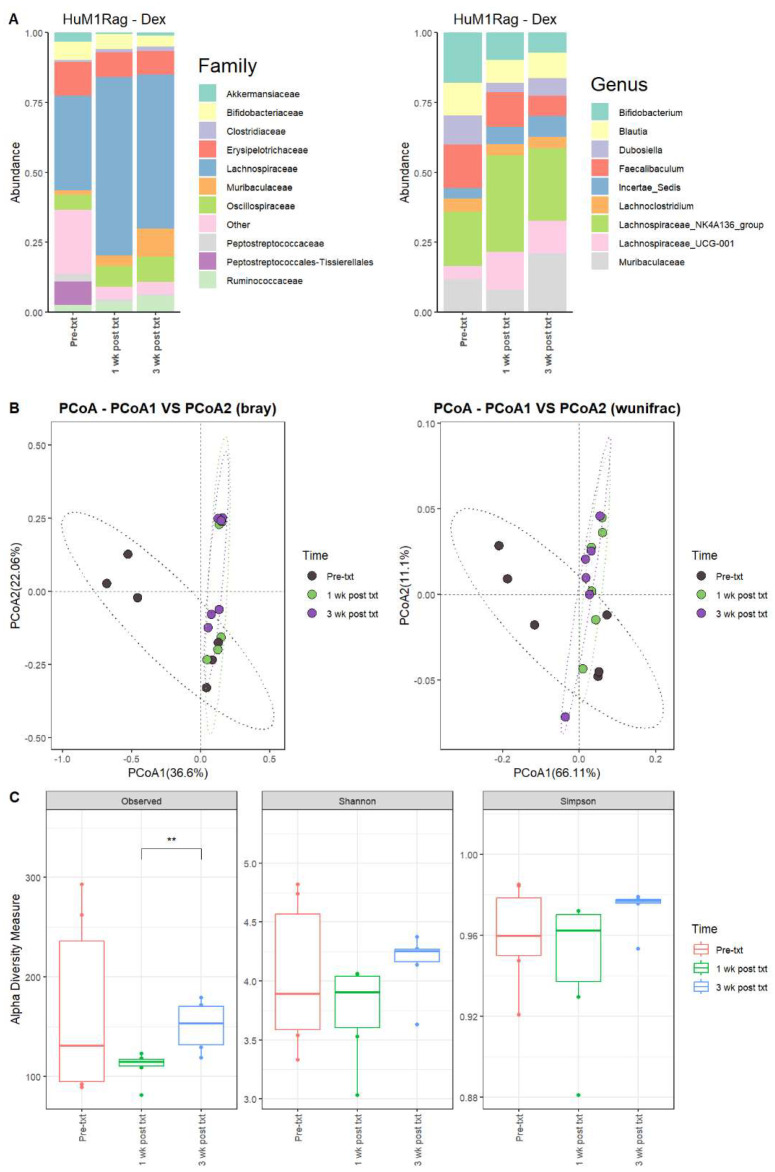
Dexamethasone significantly alters the microbiome of HuM1Rag. (**A**) The relative abundance of the top 10 taxa at the family level and Genus level across the HuM1Rag dexamethasone samples: Pre-txt, 1 wk post txt, and 3 wk post txt. (**B**) Beta diversity was determined utilizing the Bray–Curtis and weighted UniFrac metrics for the indicated groups. (**C**) Alpha-diversity measurements (observed ASVs, Shannon diversity index, and Simpson’s index) were obtained for the indicated groups.

Next, we examined the microbial changes following dexamethasone treatment in the HuM1Rag and HuM2Rag mice. In HuM1Rag mice 1 wk post txt with dexamethasone, there were overall shifts in the microbial composition. Firmicute abundance increased, while Actinobacteriota, Proteobacteria, and Verrucomicrobiota abundance decreased. Pre-treatment (Pre-txt) was characterized by a large family abundance of Lachnospiraceae (~34%) members, and there was a significant increase in the abundance of Lachnospiraceae members (~64%) 1 wk post txt (Figure 5A). At the Genus level, *Lachnospiraceae_NK4A136_group* (~20%), *Bifidobacterium* (~18%), and *Faecalibaculum* (~16%) dominated the microbial composition pre-treatment. However, at 1 wk post txt, there was a decrease in *Faecalibaculum* (~12%) and *Bifidobacterium* (~10%), accompanied by an increase in *Lachnospiraceae_NK4A136_group* (~34%) and *Lachnospiraceae_UCG-001* (~14%) (Table 4). These data support that dexamethasone altered the microbial composition in the HuM1Rag mice.

#### 3.2.8. HuM1Rag Mice Did Not Recover to Original Microbial Composition from Dexamethasone Treatment

In HuM1Rag mice 3 wk post txt, *Lachnospiraceae_NK4A136_group* (~26%) was still the dominant member of the HuM1Rag microbiota, followed by Muribaculaceae (~21%) and *Lachnospiraceae_UCG-001* (~12%) (Figure 5A; Table 4). In examining beta diversity, HuM1Rag pre-txt, 1 wk post txt, and 3 wk post txt displayed distinct clustering (Figure 5B). PERMANOVA statistics supported the significant dissimilarity among the sample groups (R^2^ = 0.21), with *p* values (<0.05), and PERMDISP revealed a significant dispersion of the samples (*p* < 0.05). Additionally, the 1 wk post txt and 3 wk post txt samples clustered together away from the pre-txt samples. Overall, this indicates that the microbial composition did not recover from the treatment with dexamethasone. Alpha diversity observed ASVs revealed significant (*p* < 0.05) differences between 1 wk post txt and 3 wk post txt in the HuM1Rag mice treated with dexamethasone (Figure 5C), with no significant change in the Shannon or Simpson diversity. The HuM1Rag mice microbiota did not recover following the dexamethasone treatment.

#### 3.2.9. HuM2Rag Showed Overall Recovery from Dexamethasone Treatment

**Figure 6:**
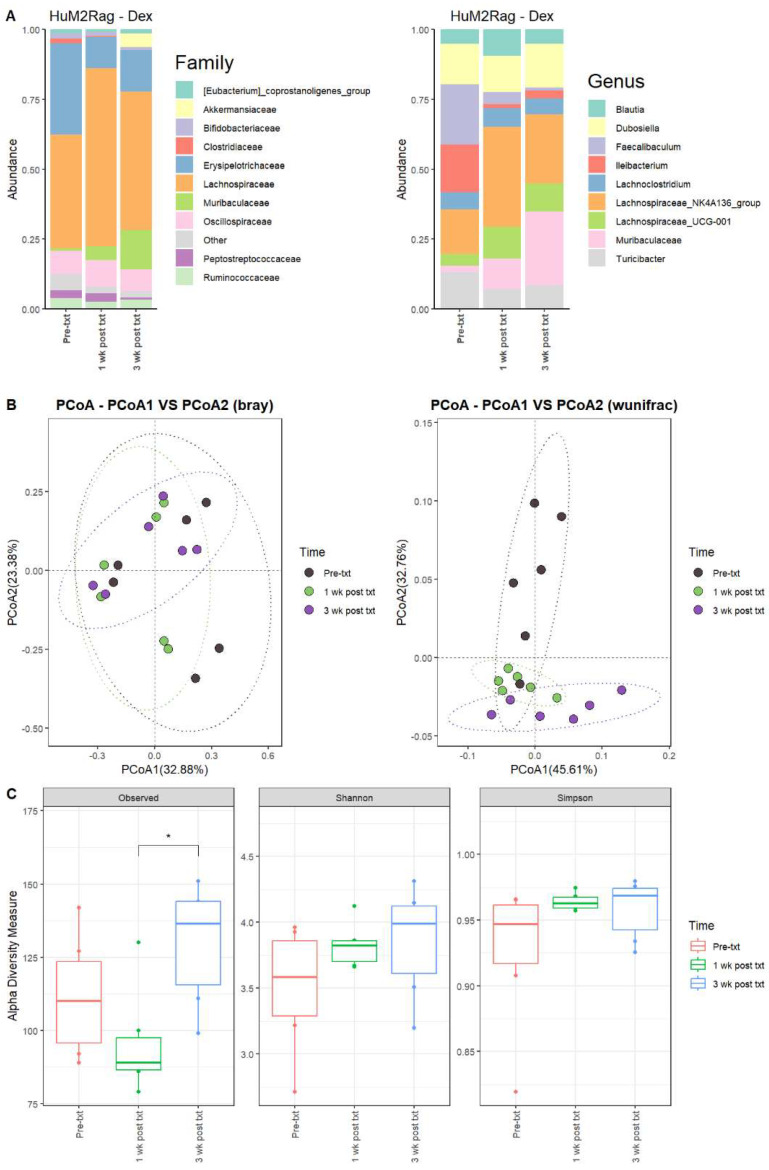
HuM2Rag showed overall recovery from the dexamethasone treatment. (**A**) The relative abundance of the top 10 taxa at the family level and Genus level across the HuM1Rag dexamethasone samples: Pre-txt, 1 wk post txt, and 3 wk post txt. (**B**) Beta diversity was determined utilizing the Bray–Curtis and weighted UniFrac metrics for the indicated groups. (**C**) Alpha-diversity measurements (observed ASVs, Shannon diversity index, and Simpson’s index) were obtained for the indicated groups.

For the HuM2Rag mice treated with dexamethasone, there were observed changes in the abundance of certain microbes. For 1 wk post txt and 3 wk post txt with dexamethasone, Firmicutes decreased in abundance, while Bacteroidota increased in overall abundance. Pre-txt was characterized by a large abundance of Lachnospiraceae (~41%) and Erysipelotrichaceae (~33%) family members, and 1 wk post txt, there was an increase in Lachnospiraceae members (~60%) and a decrease in Erysipelotrichaceae (~11%) (Figure 6A). At the Genus level, *Faecalibaculum* (~22%), *Ileibacterium* (~17%), *Lachnospiraceae_NK4A136_group* (~16%), *Dubosiella* (~14%), and *Turicibacter* (~13%) dominated the microbial composition. At 1 wk post txt, there was a decrease in *Faecalibaculum* (~4%), *Ileibacterium* (~1%), and *Turicibacter* (~7%), accompanied by an increase in *Lachnospiraceae_NK4A136_group* (~36%), Muribaculaceae (~11%), and *Lachnospiraceae_UCG-001* (~11%) (Table 5). This indicates that certain microbial groups were altered following the dexamethasone treatment.

#### 3.2.10. HuM2Rag Mice Recovered from Original Microbial Composition Post-Treatment Dexamethasone

Looking at the recovery of the microbiome, the HuM2Rag microbiome post-treatment with dexamethasone more closely resembled the pre-txt composition and diversities. At 3 wk post txt, Muribaculaceae (~26%) and *Lachnospiraceae_NK4A136_group* (~25%) became the dominant members of the HuM2Rag microbiota, followed by *Dubosiella* (~16%) (Figure 6A; Table 5). In examining beta diversity, the HuM2Rag mice resulted in a distinct clustering of pre-txt, 1 wk post txt, and 3 wk post txt groups (Figure 6B). PERMANOVA statistics supported the significant dissimilarity among the sample groups (R^2^ = 0.18), with *p* values (< 0.05), and PERMDISP revealed no significant dispersion of the samples (*p* > 0.05). Additionally, the 1 wk post txt and 3 wk post txt samples clustered together with the pre-txt samples, indicating that the microbial composition recovered from the treatment. The HuM2Rag mice revealed significant (*p* < 0.05) differences in alpha diversity observed ASVs between 1 wk post txt and 3 wk post txt with dexamethasone (Figure 6C). These data suggest that the microbiota recovered from the dexamethasone treatment in the HuM2Rag mice.

#### 3.2.11. Predicted Functional Analysis of HuM1Rag Revealed Significant Pathways Affected by Dexamethasone Treatment

**Figure 7:**
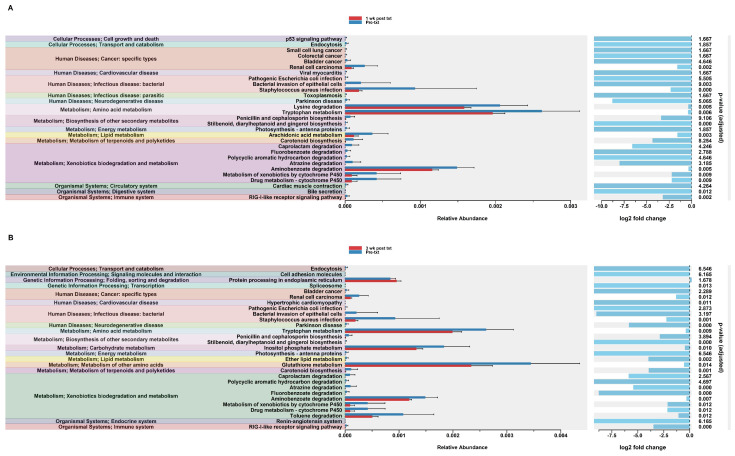
Predicted functional analysis of HuM1Rag revealed significant pathways affected by the dexamethasone treatment. Predicted functional pathways were determined via PICRUSt and displayed are the differential abundance of the predicted functional pathways. (**A**) HuM1Rag Pre-txt vs. 1 wk post txt and (**B**) HuM1Rag Pre-txt vs. 3 wk post txt.

In order to assess potential functional differences following the dexamethasone treatment, PiCrust2 analyses were performed. PiCrust2 analysis revealed significant differences in the predicted KEGG pathways at 1 wk post txt with dexamethasone in the HuM1Rag mice (Figure 7A). There was a significant down-regulation in amino acid metabolism, lipid metabolism, xenobiotic biodegradation and metabolism, the biosynthesis of secondary metabolites, bile secretion, and RIG-1-like receptor signaling. Additionally, a significant down-regulation in the pathways associated with cancer and infectious disease was observed. At 3 wk post txt with dexamethasone in the HuM1Rag mice, a down-regulation in the same pathways as 1-wk post txt was noted (Figure 7B). Additionally, a significant down-regulation in the pathways associated with transcription, cardiovascular disease, and neurodegenerative diseases was observed. This indicates that numerous pathways are predicted to be altered in the HuM1Rag mice following the dexamethasone treatment.

#### 3.2.12. Predicted Functional Analysis of HuM2Rag Revealed Dexamethasone Treatment Had Fewer Pathways Altered Compared to HuM1Rag Mice

**Figure 8:**
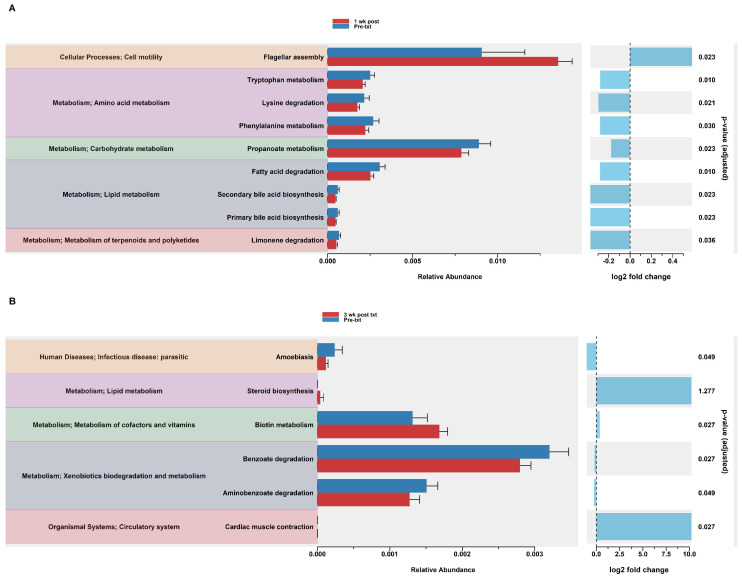
Predicted functional analysis of HuM2Rag revealed the dexamethasone treatment had fewer pathways altered compared to the HuM1Rag mice. Predicted functional pathways were determined via PICRUSt and displayed are the differential abundance of the predicted functional pathways. (**A**) HuM2Rag Pre-txt vs. 1 wk post txt and (**B**) HuM2Rag Pre-txt vs. 3 wk post txt.

In the HuM2Rag mice treated with dexamethasone, PiCrust2 analysis revealed a significant upregulation in flagellar assembly and a down-regulation in the pathways associated with the metabolism of amino acids, carbohydrates, lipids, terpenoids, and polyketides at 1 wk post txt (Figure 8A). At 3 wk post txt, there was a significant increase in biotin metabolism and muscle contraction. Additionally, a significant down-regulation in the pathways associated with infectious disease and xenobiotic biodegradation was observed (Figure 8B). Overall, the PiCrust2 analyses revealed that the HuM1Rag mice exhibited many more predicted pathway changes following the dexamethasone treatment than the the HuM2Rag mice.

## 4. Discussion

Dexamethasone and TMP-SMX are two medications commonly prescribed alongside standard-of-care chemo- and radiotherapy for patients with GBM to decrease cerebral edema and prevent bacterial infections, respectively. Since the gut microbiota can influence the efficacy of anti-tumor therapy, it is important to understand how routine medications prescribed during treatment can affect the gut microbiota of patients with GBM. Interestingly, a study in laboratory mice treated with TMP-SMX orally for 14 days found no notable changes in the taxonomic composition of the mouse microbiota [44]. In humans with short bowel syndrome, TMP-SMX treatment did impact the microbiota leading to an increase in Proteobacteria and a decrease in Firmicute members [45].

Another concern with TMP-SMX treatment is the risk of developing antibiotic-resistant infections that can influence the systemic immune response and thus anti-tumor response. The chronic use of TMP-SMX in children being treated for ear infections has been shown to lead to drug-resistant Enterobacteriaceae in the gut microbiota [46]. While dexamethasone is not an antibiotic such as TMP-SMX, it is a potent glucocorticoid and may alter the gut microbiota through endocrine and immune mechanisms. Previous studies of mice treated with dexamethasone have shown a decrease in alpha diversity in the colon microbiota accompanied by an increase in the Firmicutes phyla and Lachnospiraceae family abundance [47]. Interestingly, in rabbits and rats, dexamethasone treatment has been shown to decrease Firmicute abundance [48,49]. These studies suggest that variations in baseline gut microbial compositions may result in unique shifts in microbial populations in response to TMP-SMX and dexamethasone treatment, and may affect overall host health; for this reason, it is important to investigate microbial response utilizing a humanized microbiome model.

At a phylum level, the HuM1Rag and HuM2Rag mice displayed similarities in microbial composition pre-txt, being primarily composed of Firmicute members. Differences were notably observed at a family and Genus level as HuM1Rag revealed to be primarily composed of *Lachnospiraceae_NK4A136_group*, *Lactobacillus*, and *Faecalibaculum*. In contrast, the HuM2Rag mice were primarily composed of *Lachnospiraceae_NK4A136_group*, *Ileibacterium*, *Dubosiella*, and *Turicibacter*. These differences initially present may contribute to the overall structure and function of the microbiome, as variations in carbohydrate, amino acid, energy, cofactor, and vitamin metabolism were significantly different at the baseline between the HuM1Rag and HuM2Rag mice. Therefore, based on these initial differences, perturbations via TMP-SMX and dexamethasone treatment may alter the HuM1Rag and HuM2Rag microbial ecosystem, affecting functionality, structure, and recovery [50,51]. Previously noted recovery from an antibiotic regime is dependent on the individual, which indicates that the baseline microbiota present in the HuM1Rag and HuM2Rag mice will affect microbial resistance and recovery [50,52,53]. Furthermore, TMP-SMX is a broadscale antibiotic that may affect Gram-negative and Gram-positive bacteria; however, depending on the bacterial structure, function, and location, TMP-SMX may not affect all communities present in the HuM1Rag and HuM2Rag mice [54]. In contrast, dexamethasone is a corticosteroid, which has been observed to affect taxonomic composition, and an upregulation of Muc2 gene expression, which has been linked to a proinflammatory gut environment [55].

The HuM1Rag mice 1 wk post txt with TMP-SMX demonstrated microbial community shifts. Decreases in the populations of *Lachnospiraceae_NK4A136_group* and *Blautia* occurred, and an increase in the relative abundance of *Bifidobacterium*, *Dubosiella*, *Turicibacter*, and Muribaculaceae members was observed. These results demonstrate that TMP-SMX affected the microbial composition; however, no adverse phenotypic effects were observed in the HuM1Rag mice, nor do these shifts indicate pathogenic microbial members or dysbiosis [56]. An increase in *Dubosiella* members has been linked to anti-aging, and improving obesity, hypertension, and liver disease; additionally, it is linked to promoting beneficial microbial taxon such as *Bifidobacterium*, which was observed in our data [57]. *Bifidobacterium* has potential beneficial therapeutic effects, such as antimicrobial and immunomodulatory effects via increasing immunoglobulins and inducing or reducing pro- or anti-inflammatory cytokines [58].

Previous studies have revealed that the gut microbiota plays a major role in cancer microenvironments, affecting tumor response to immune checkpoint inhibitors [59,60,61]. For HuM1Rag 1 wk post txt with TMP-SMX there was an increase in the overall relative abundance of *Turicibacter*, which has been linked to successful anti-PD-1 treatment albeit with concurrent immune-related adverse events [62]. On the contrary, a decrease in *Blautia* populations was observed 1 wk post txt, and larger abundances of *Blautia* have been linked to successful immunotherapy without immune-related adverse events [62]. The HuM1Rag mice 1 wk post txt exhibited a significant upregulation in primary and secondary bile acid synthesis in contrast to pre-treatment. *Turicibacter* members contribute to bile acid mediation and modification, which may affect immunotherapy. Previous studies have revealed that primary and secondary bile acids such as ursodeoxycholic acid have been linked to successful or unsuccessful immunotherapy treatments [63,64,65]. Additionally, changes in bile acid production can potentially lead to dysbiosis and may make patients susceptible to infection by exogenous, or opportunistic microbes present in the gut [66], which can affect overall patient recovery.

In the HuM1Rag mice 3 wk post-treatment with TMP-SMX, Muribaculaceae members dominated the microbial community, a noticeable difference from the original abundance of *Faecalibaculum*, *Lachnospiraceae_NK4A136_group*, and *Blautia* pre-txt. These results indicate that the microbiome may have not recovered from the TMP-SMX treatment, as the microbial composition has significantly changed which was supported via alpha and beta diversity, and predicted functional analysis resulted in changes in membrane transport, carbohydrate metabolism, and other secondary metabolites. The Muribaculaceae population may be a benefit to overall host health as Muribaculaceae is associated with improved mucus integrity [67,68]. Improved mucus integrity would limit microbe–immune cell interactions and mitigate the potential negative effects of dysbiosis on the host immune system. While metabolomic analysis was not performed due to experimental constraints, the upregulation in the pathways associated with carbohydrate metabolism 3 wk post txt could suggest changes in short-chain fatty acid (SCFA) synthesis. Different SCFAs are known to impact the host immune system in varying ways, but in general, they are thought to promote an anti-inflammatory and tolerogenic phenotype in immune cells through inhibiting histone deacetylases [69,70,71].

Regarding the HuM2Rag mice treated with TMP-SMX, 1 wk post-txt displayed decreases in *Turicibacter*, and *Faecalibaculum*, and increases in *Lachnospiraceae_NK4A136_group*, *Dubosiella*, and Muribaculaceae. Shifts in relative abundance were observed in the HuM2Rag mice at treatment time points; however, alpha diversity revealed no significant difference between pre-txt, 1 wk post txt, and 3 wk post txt. This potentially indicates that TMP-SMX did not significantly affect microbial communities between time points. Beta diversity did support a low significant effect of the treatment group on microbial composition; however, the HuM1Rag mice revealed significant differences across all diversity metrics across all time points. Furthermore, the functional pathway analysis resulted in a single pathway difference between pre-txt and 1 wk post txt in the HuM2Rag mice in contrast to the HuM1Rag mice treated with TMP-SMX which resulted in more overall pathways affected via TMP-SMX; likewise, 3 wk post txt compared to pre-txt, only four pathways were affected in the HuM2Rag mice. The ability of the microbiome to maintain structure and function in response to structural and environmental perturbations may be key to overcoming antibiotic treatment, diseases, and long-term treatment plans that patients must endure [72,73].

The effect of dexamethasone 1 wk post-treatment in the HuM1Rag mice resulted in decreases in *Bifidobacterium*, *Faecalibaculum*, *Blautia*, Muribaculaceae, and increases in *Lachnospiraceae_NK4A136_group*, and *Lachnospiraceae_UCG-001*. Previous studies have revealed that intaking *Bifidobacteria*, therefore, increasing *Bifidobacterium* abundance, resulted in a decrease in Lachnospiraceae members, which was also observed in our study [73]. A decrease in *Bifidobacterium* may negatively impact patient outcomes, as members of this Genus have been linked to gut integrity, immune modulation, and SCFA production [74,75,76]. *Bifidobacterium* members have also been linked to successful cancer therapies, oxaliplatin, and PD-1 blockade, as transcriptomic analysis revealed the linkage between *Bifidobacterium* members with lymphocyte-mediated anti-cancer immunity [19,58,76]. Additionally, *Bifidobacterium* members have been linked to tryptophan metabolism, and notably 1 wk post txt tryptophan levels dropped. Interestingly, tryptophan metabolism by IDO1 has been linked to the inhibition of T cell and NK cell proliferation, so changes in tryptophan metabolism may impact the host’s immune response and thus immunotherapy efficacy [77,78].

Additionally, in the HuM1Rag treated with dexamethasone, *Lachnospiraceae NK4A136* increased. The members of *Lachnospiraceae NK4A136* play a role in colonization resistance via the production of Lantibiotics, as well as being a contributor to SCFA production. Lachnospiraceae members are known to be major butyrate producers, which acts as the energy source for colonocytes, as well as controlling gut inflammatory processes and immune system maturation [79]. This is supported as the functional pathways associated with amino acid and xenobiotics degradation metabolism were down-regulated. Additionally, 3 wk post txt tryptophan metabolism, xenobiotics degradation metabolism did not recover. This could potentially indicate that there may be potential long-term effects of dexamethasone [13,47].

HuM2Rag dexamethasone-treated mice 1 wk post txt demonstrated decreases in *Turicibacter*, *Faecalibaculum*, and *Ileibacterium* and an increase in *Lachnospiraceae_NK4A136_group* and *Lachnospiraceae_UCG-001*. At 3 wk post txt, Muribaculaceae was the dominant member, followed by *Lachnospiraceae_NK4A136_group*. Shifts in relative abundance were observed in the HuM2Rag mice at all treatment time points; however, alpha diversity revealed a significant difference in the observed ASVs, from 1 wk post txt to 3 wk post txt, which indicates that there were minimal changes in the overall alpha diversity, as Shannon and Simpson’s diversity was not significant. Additionally, beta diversity did support a low significant effect of the treatment groups on the microbial composition; however, the HuM1Rag mice treated with TMP-SMX or dexamethasone revealed significant differences across all diversity metrics across all time points. Furthermore, the functional pathway analysis resulted in nine pathways that differed between pre-txt and 1 wk post txt in the HuM2Rag mice in contrast to the HuM1Rag mice treated with dexamethasone which resulted in more overall pathways affected; likewise, 3 wk post txt compared to pre-txt, only six pathways were affected in HuM2Rag mice. This trend was also observed in the HuM2Rag mice treated with TMP-SMX. This suggests that the HuM2Rag mice microbiota may exhibit a greater resilience compared to the HuM1Rag microbiota, given that the impact of TMP-SMX and dexamethasone on microbial composition was notably less.

## 5. Conclusions

The HuM1Rag and HuM2Rag mice pre-treatment revealed differences in the microbial composition. After 1 week of TMP-SMX or dexamethasone treatment, both the HuM1Rag mice and HuM2Rag mice displayed unique responses via shifts in alpha and beta diversity. The HuM2Rag mice resulted in minimal changes in alpha and beta diversity, in contrast to the HuM1Rag mice which was also observed in predicted functional analysis, with the HuM2Rag mice revealing few pathways affected by both treatments. A limitation of this study was that the HuM1Rag and HuM2Rag mice were not treated with both TMP-SMX and dexamethasone, which can occur in patients with GBM. Additionally, functional pathway analysis was not validated with metabolomics. These data provide researchers with a further understanding of the routine medications used in the treatment of patients with the microbiome, which can affect overall treatment, health outcomes, and overall metabolic homeostasis. Perturbed microbiomes may lead to inflammation, and alter immune response and metabolic function; therefore, potential therapies using nutrition, probiotics, or prebiotics to alter the microbiome pre-treatment or during treatment may lead to an overall improved cancer therapy.

## Figures and Tables

**Table 1:** Top 10 ASVs up to a Genus level across the mean bacterial abundance for HuM1Rag and HuM2Rag.

ASV	Hum1Rag (*n* = 17)	Hum2Rag (*n* = 17)
*Lachnospiraceae_NK4A136_group*	18% ± 11%	18% ± 13%
*Lactobacillus*	17% ± 16%	3% ± 7%
*Faecalibaculum*	15% ± 13%	14% ± 26%
*Bifidobacterium*	13% ± 6%	4% ± 3%
*Blautia*	13% ± 12%	5% ± 4%
*Dubosiella*	12% ± 9%	17% ± 10%
*Turicibacter*	8% ± 6%	16% ± 10%
*Romboutsia*	3% ± 3%	5% ± 4%
*Ileibacterium*	1% ± 1%	17% ± 11%

**Table 2:** Top 10 ASVs up to a Genus level across the mean bacterial abundance of HuM1Rag for the TMP–SMX treatment.

ASV	Pre-txt (*n* = 7)	1 wk Post txt (*n* = 7)	3 wk Post txt (*n* = 7)
*Faecalibaculum*	17% ± 13%	16% ± 20%	7% ± 6%
*Lachnospiraceae_NK4A136_group*	15% ± 13%	5% ± 3%	16% ± 11%
*Blautia*	14% ± 15%	2% ± 1%	4% ± 2%
*Dubosiella*	13% ± 10%	18% ± 8%	14% ± 9%
*Bifidobacterium*	11% ± 3%	19% ± 8%	8% ± 4%
*Lactobacillus*	11% ± 11%	12% ± 6%	13% ± 6%
*Turicibacter*	9% ± 7%	15% ± 13%	2% ± 2%
*Muribaculaceae*	6% ± 8%	12% ± 3%	25% ± 8%
*Lachnospiraceae_UCG-001*	5% ± 3%	3% ± 2%	11% ± 10%

**Table 3:** Top 10 ASVs up to a Genus level across the mean bacterial abundance of HuM2Rag for the TMP-SMX treatment.

ASV	Pre-txt (*n* = 6)	1 wk Post txt (*n* = 6)	3 wk Post txt (*n* = 6)
*Turicibacter*	24% ± 14%	11% ± 3%	12% ± 7%
*Faecalibaculum*	21% ± 31%	4% ± 3%	2% ± 2%
*Lachnospiraceae_NK4A136_group*	14% ± 9%	24% ± 12%	16% ± 11%
*Ileibacterium*	14% ± 11%	3% ± 6%	3% ± 2%
*Dubosiella*	14% ± 11%	21% ± 9%	22% ± 9%
*Blautia*	5% ± 3%	5% ± 2%	4% ± 3%
*[Eubacterium]_xylanophilum_group*	3% ± 2%	11% ± 7%	2% ± 2%
*Muribaculaceae*	3% ± 7%	19% ± 8%	33% ± 6%
*Bifidobacterium*	3% ± 3%	2% ± 2%	6% ± 5%

**Table 4:** Top 10 ASVs up to a Genus level across the mean bacterial abundance of HuM1Rag for the dexamethasone treatment.

ASV	Pre-txt (*n* = 6)	1 wk Post txt (*n* = 6)	3 wk Post txt (*n* = 6)
*Lachnospiraceae_NK4A136_group*	20% ± 8%	34% ± 19%	26% ± 10%
*Bifidobacterium*	18% ± 10%	10% ± 9%	7% ± 7%
*Faecalibaculum*	16% ± 15%	12% ± 14%	7% ± 6%
*Muribaculaceae*	12% ± 14%	8% ± 6%	21% ± 8%
*Blautia*	12% ± 8%	8% ± 5%	9% ± 5%
*Dubosiella*	10% ± 10%	3% ± 2%	6% ± 3%
*Lachnospiraceae_UCG-001*	5% ± 4%	14% ± 10%	12% ± 6%
*Lachnoclostridium*	5% ± 2%	4% ± 2%	4% ± 2%
*Incertae_Sedis*	4% ± 3%	6% ± 2%	7% ± 2%

**Table 5:** Top 10 ASVs up to a Genus level across the mean bacterial abundance of HuM2Rag for the dexamethasone treatment.

ASV	Pre-txt (*n* = 6)	1 wk Post txt (*n* = 6)	3 wk Post txt (*n* = 6)
*Faecalibaculum*	22% ± 33%	4% ± 4%	1% ± 1%
*Ileibacterium*	17% ± 16%	1% ± 2%	3% ± 3%
*Lachnospiraceae_NK4A136_group*	16% ± 12%	36% ± 13%	25% ± 16%
*Dubosiella*	14% ± 12%	13% ± 11%	16% ± 13%
*Turicibacter*	13% ± 8%	7% ± 3%	8% ± 3%
*Lachnoclostridium*	6% ± 4%	7% ± 1%	6% ± 5%
*Blautia*	5% ± 4%	9% ± 6%	5% ± 4%
*Lachnospiraceae_UCG-001*	4% ± 4%	11% ± 11%	10% ± 9%
*Muribaculaceae*	2% ± 5%	11% ± 9%	26% ± 17%

## Data Availability

The high-throughput amplicon sequencing data of all mice samples are publicly available on the BioSample Submission Portal (https://www.ncbi.nlm.nih.gov/bioproject/PRJNA1100598, accessed on 9 April 2024) under the BioProject ID PRJNA1100598.

## Supplementary Materials

The following supporting information can be downloaded at: https://www.mdpi.com/article/10.3390/microorganisms12051015/s1, Figure S1: ASV Distribution Across Samples, Figure S2: HuM1Rag Vehicle treatment, Figure S3: HuM2Rag Vehicle treatment, Figure S4: HuM1Rag and HuM2Rag Functional Pathways Pre-Treatment, Data S1: DADA2, Data S2: ASV Table.
