# Supplementary figures and images for: Exploring Gut Microbiota Alterations with Trimethoprim-Sulfamethoxazole and Dexamethasone in a Humanized Microbiome Mouse Model
Source: Microorganisms. 2024 May 17;12(5):1015. doi: 10.3390/microorganisms12051015 (PMC11124107; doi:10.3390/microorganisms12051015)

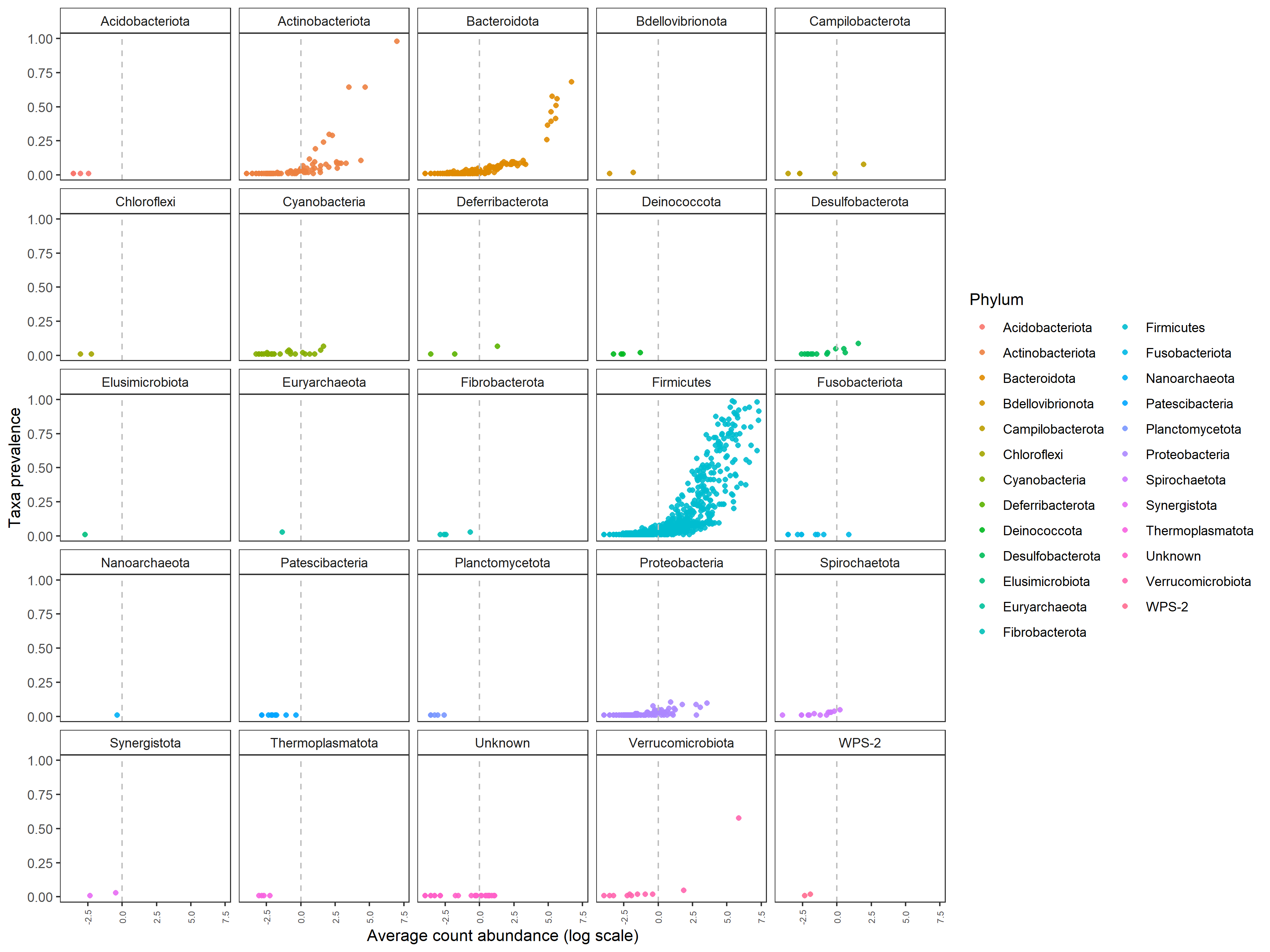

Supplement: Supplementary file 1 [file microorganisms-12-01015-s001.zip › Supplementary Figure S1.tiff]

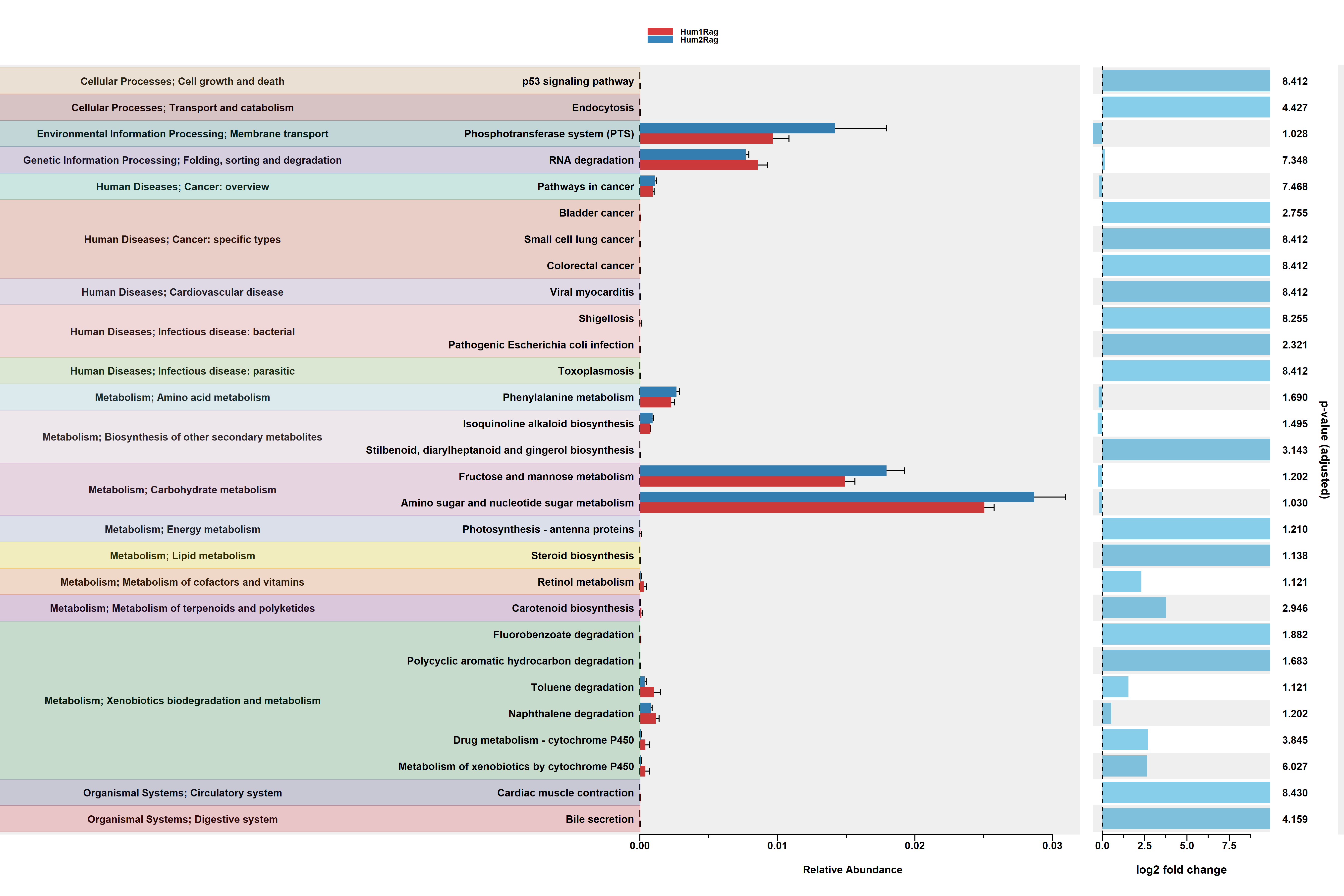

Supplement: Supplementary file 1 [file microorganisms-12-01015-s001.zip › Supplementary Figure S2.tiff]

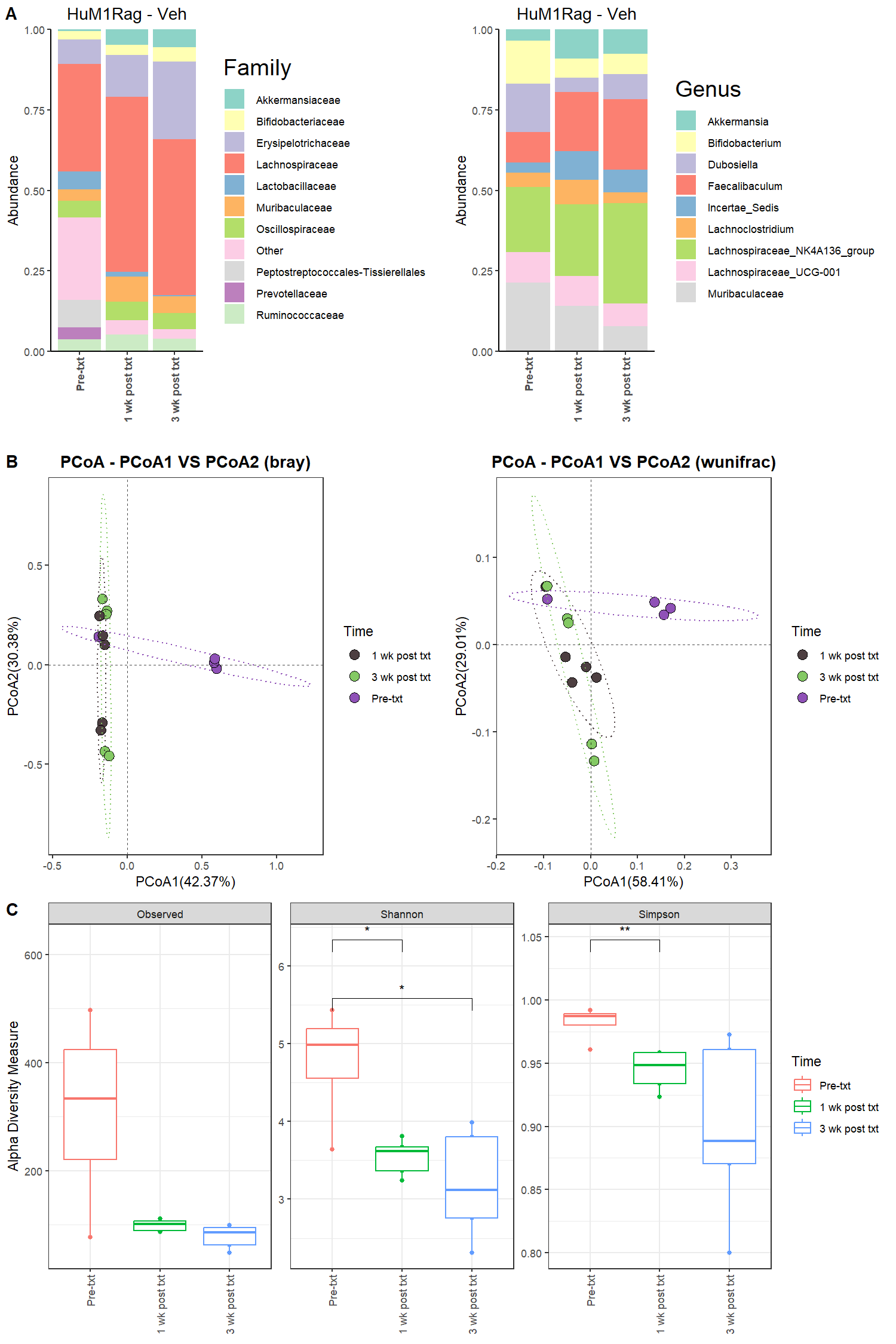

Supplement: Supplementary file 1 [file microorganisms-12-01015-s001.zip › Supplementary Figure S3.tiff]

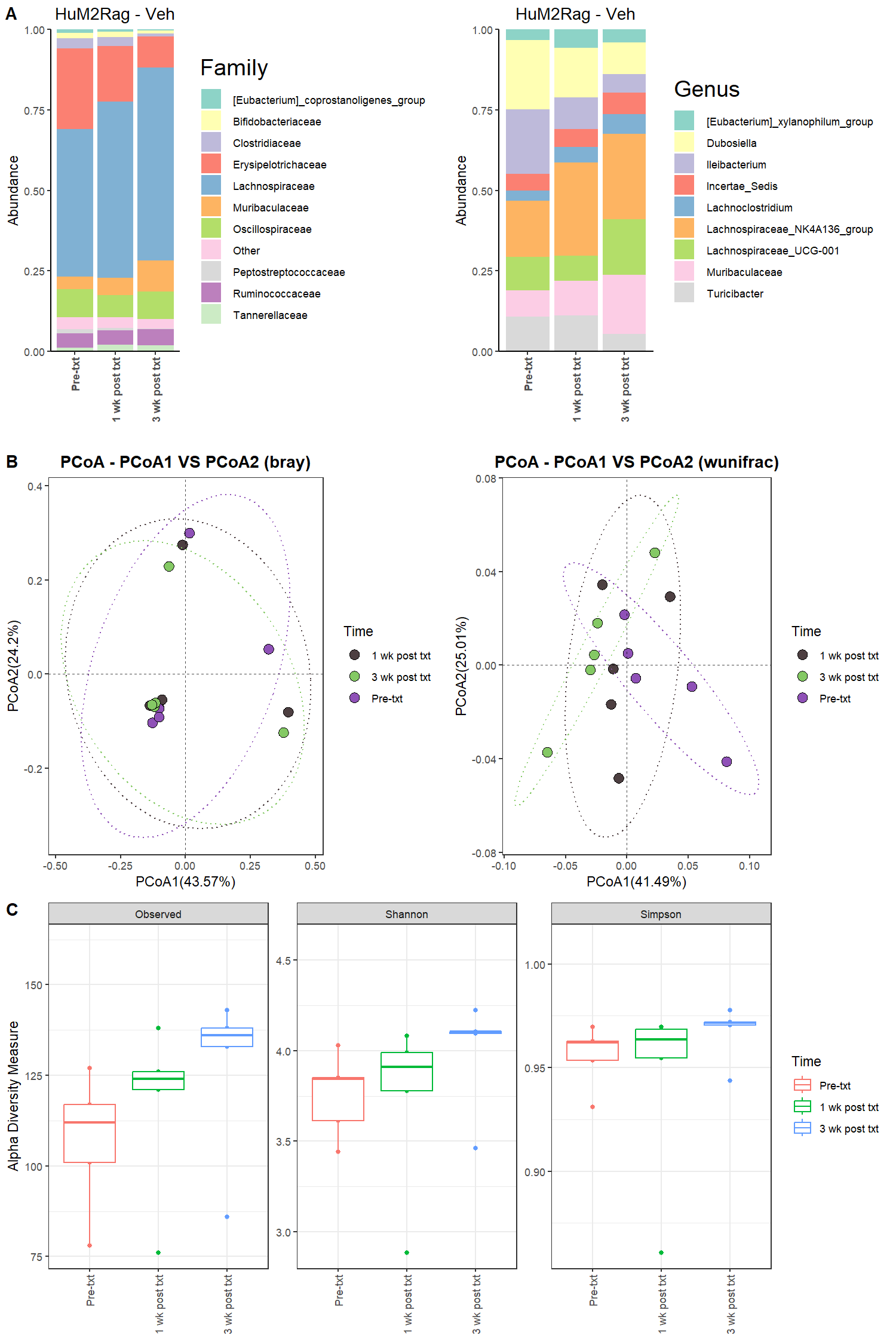

Supplement: Supplementary file 1 [file microorganisms-12-01015-s001.zip › Supplementary Figure S4.tiff]
